# Supplementary figures and images for: Dual function of mitochondrial complex III in Plasmodium falciparum
Source: PLoS One. 2026 Feb 20;21(2):e0334727. doi: 10.1371/journal.pone.0334727 (PMC12923049; doi:10.1371/journal.pone.0334727)

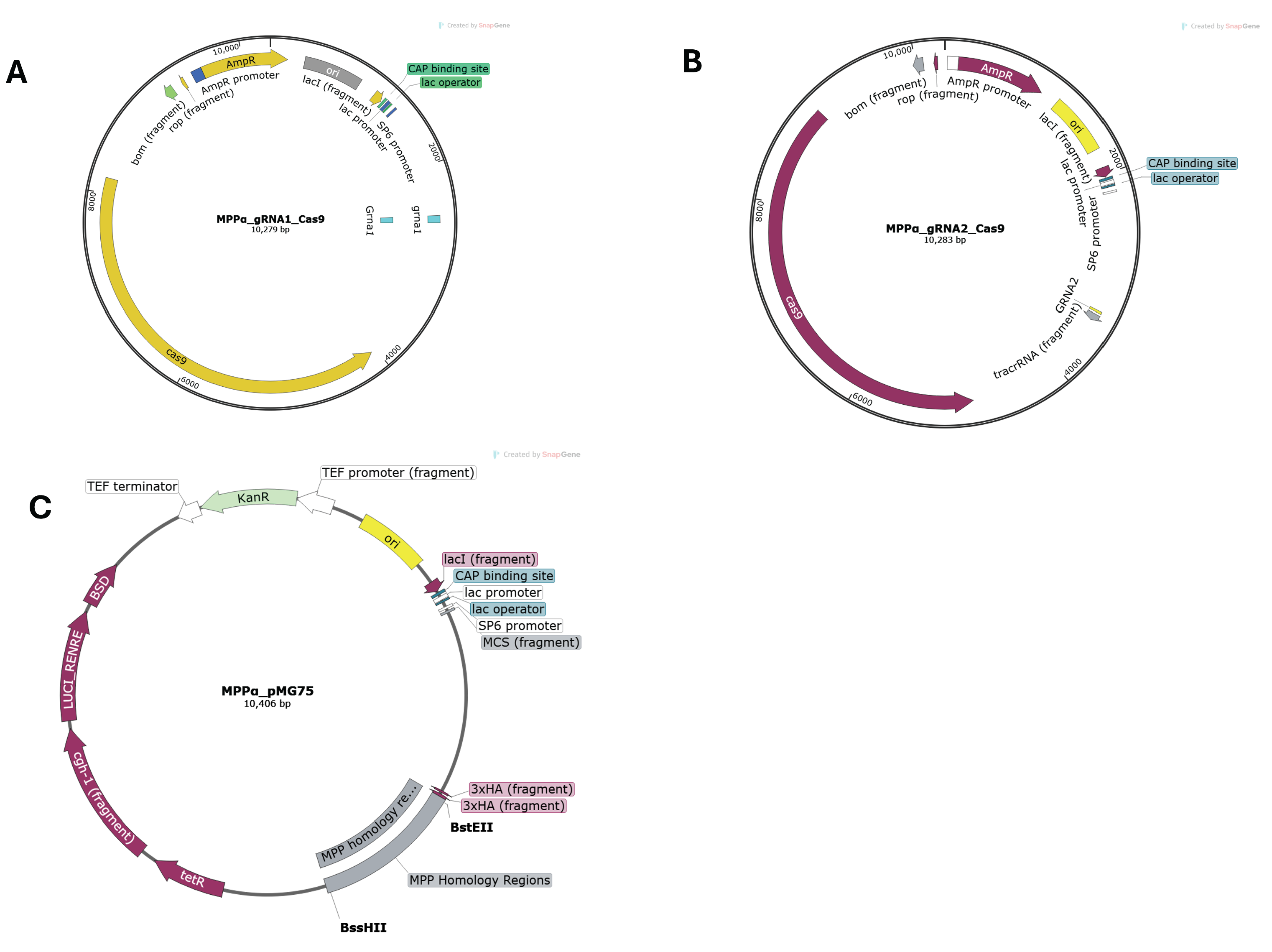

Supplement: S1 Fig — Maps of the two gRNA plasmids (A) and (B), and the map for double cross over modification of MPPα locus (C) are shown. (TIFF) [file pone.0334727.s001.tiff]

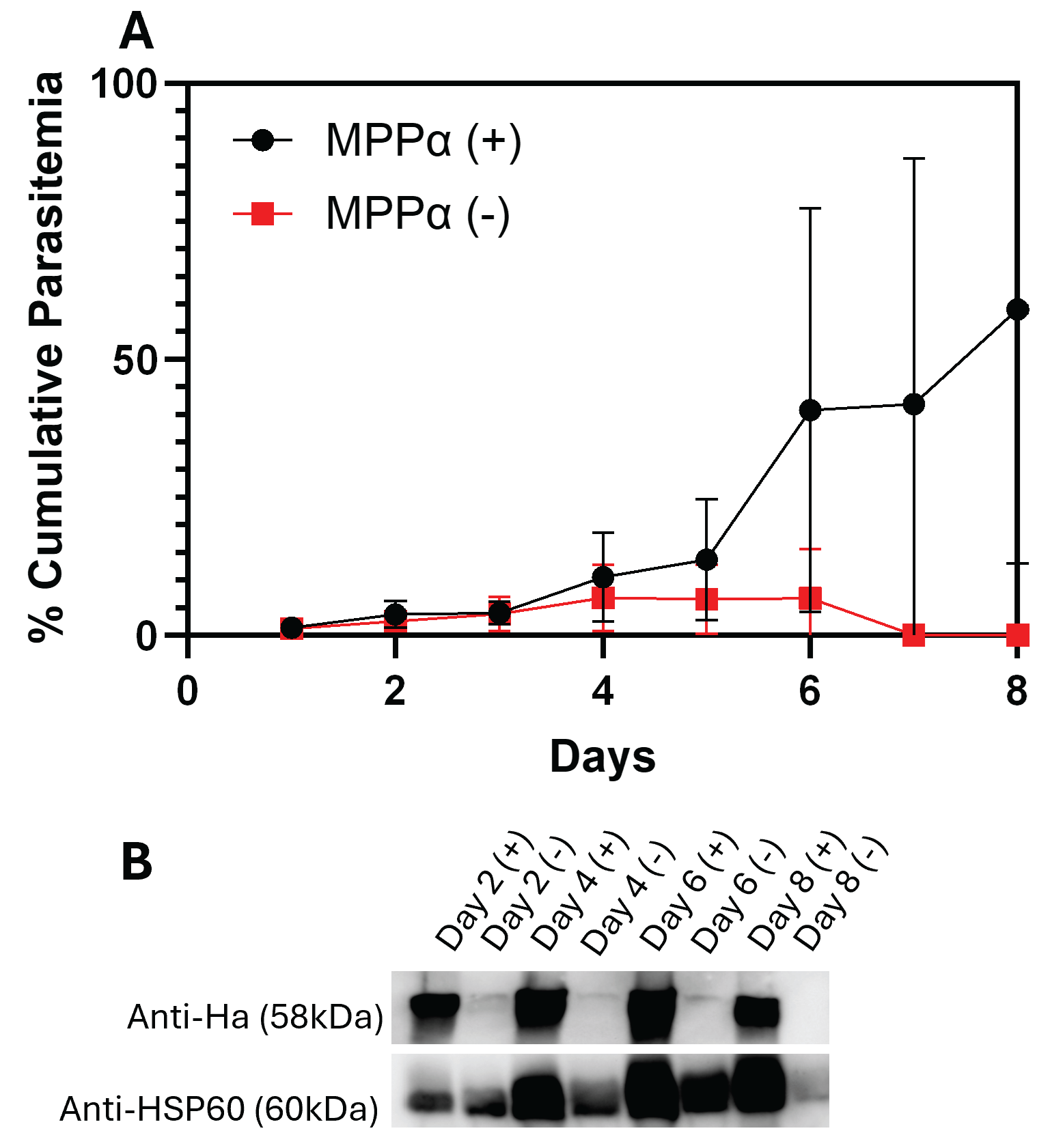

Supplement: S2 Fig — (A) Assessment of growth of MPPα + and MPPα - parasites in Dd2attb parasites. Growth assay was conducted in triplicate by counting 500 parasites via Giemsa stain. (B) Western blot analysis using anti-HA and anti-Aldolase antibodies. (TIFF) [file pone.0334727.s002.tiff]

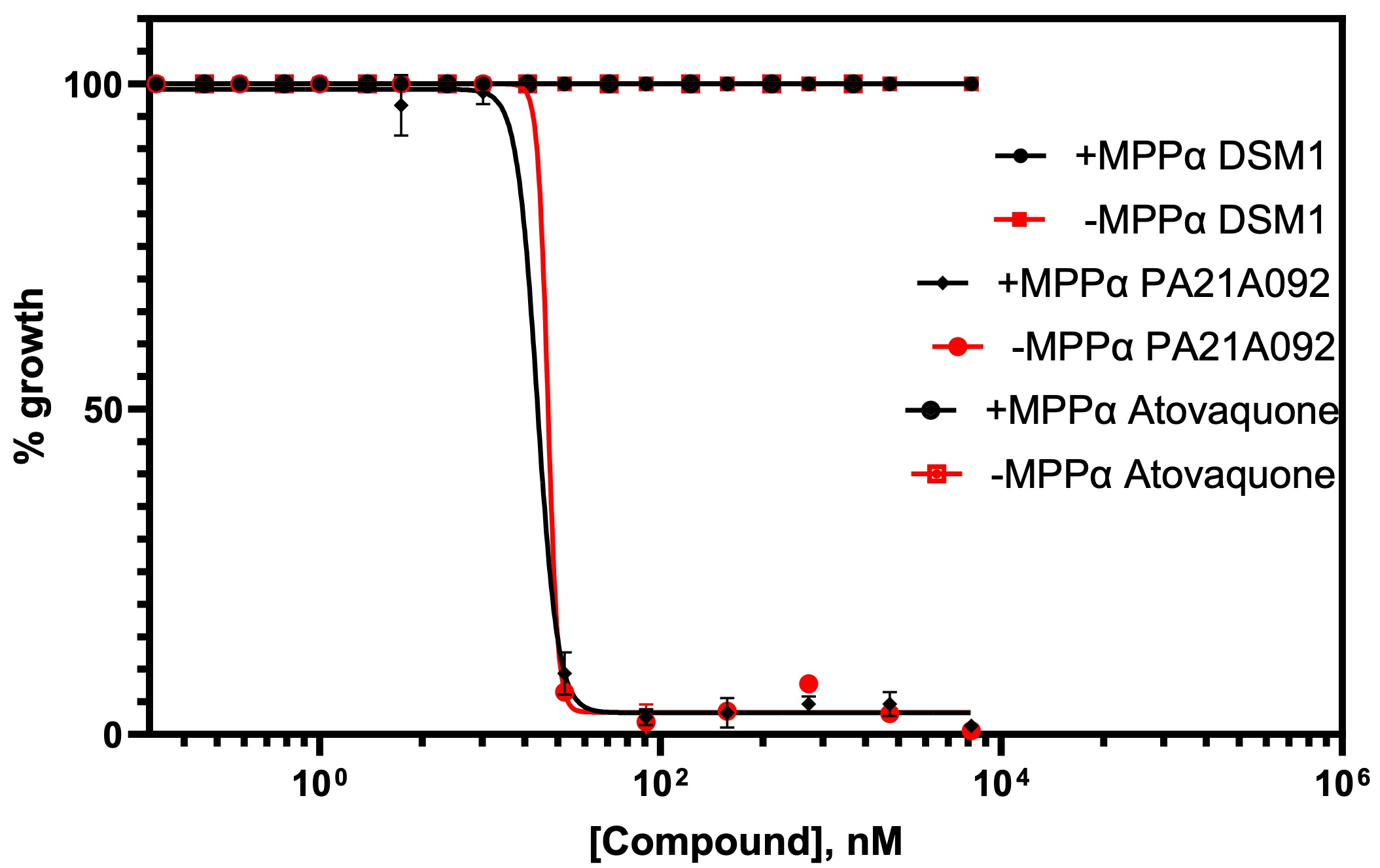

Supplement: S3 Fig — Parasite growth inhibition assessed by 3H-hypoxanthine incorporation following DSM1, atovaquone, PA21A092 treatment. For MPPα - parasites, aTc was removed for 48 h followed by 3H-hypoxanthine addition for 72 hr. (TIFF) [file pone.0334727.s003.tiff]

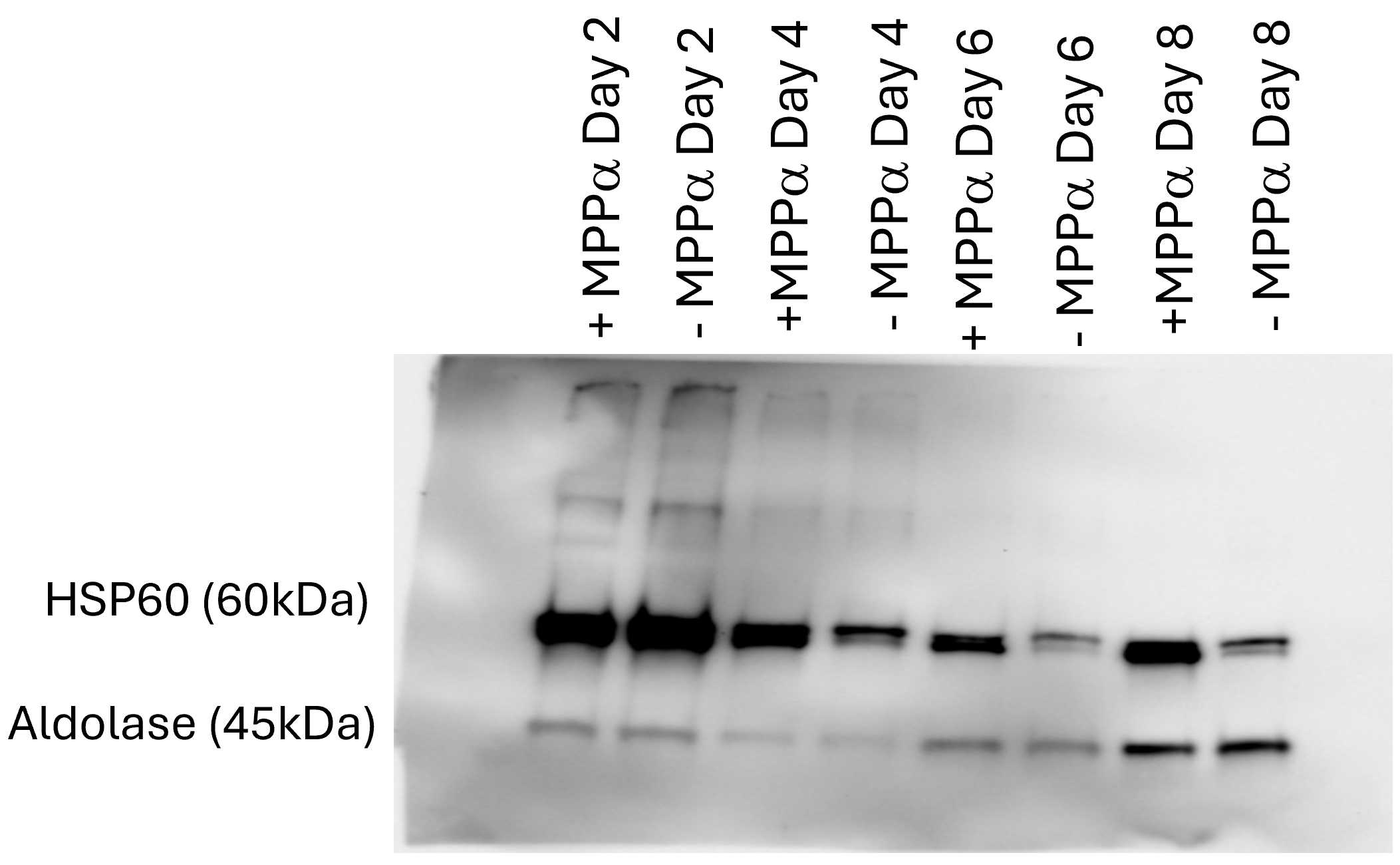

Supplement: S4 Fig — Western blot analysis of MPPα+ and MPPα- parasites using anti-HSP60 and anti-Aldolase antibodies. As MPPαdepletion progresses, more of the unprocessed HSP60 band is present in the Western Blot analysis. (TIF) [file pone.0334727.s004.tif]

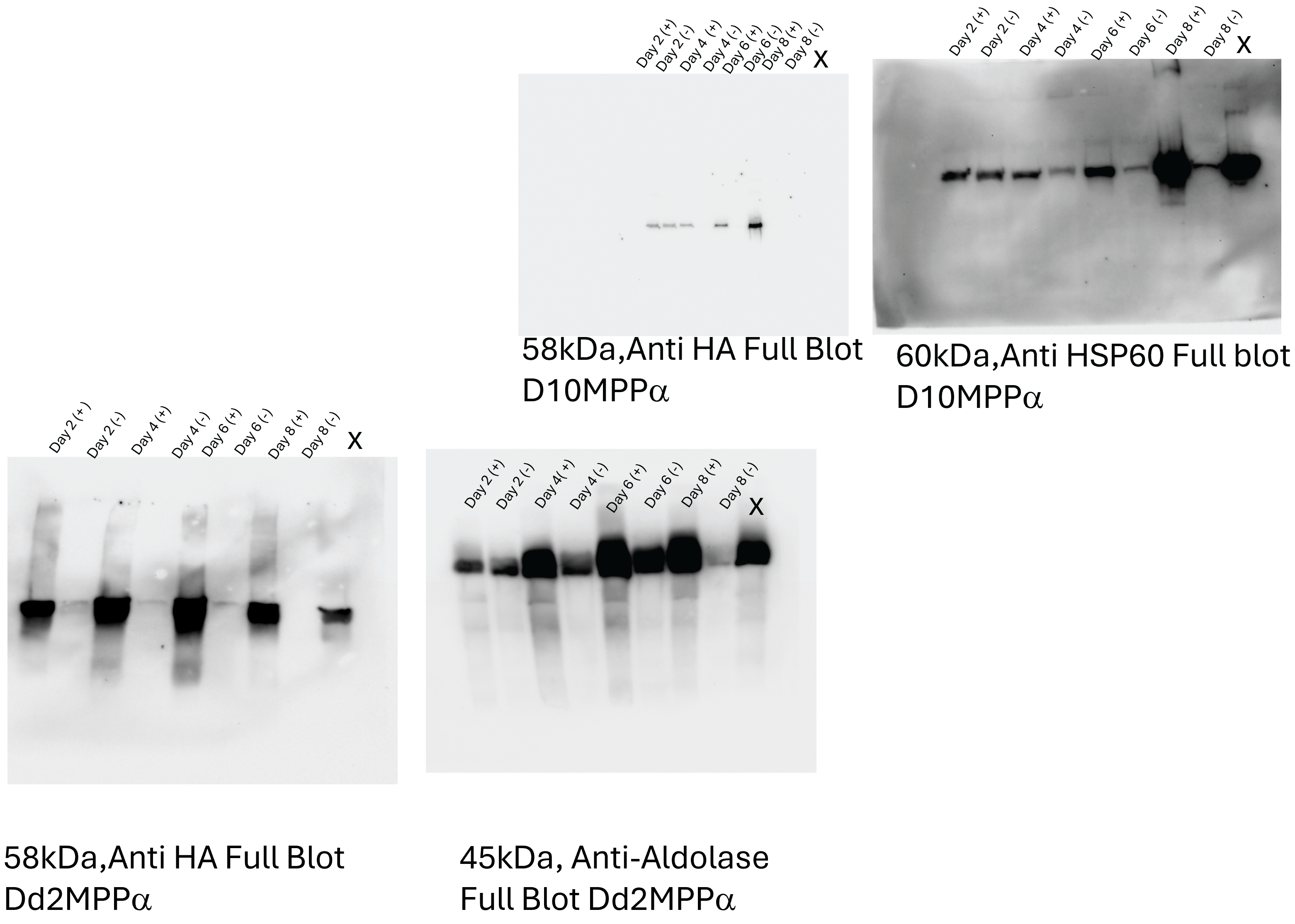

Supplement: S2 File — Uncropped and unchanged images that correspond to the cropped western blots found in Fig 2 and S2 of the manuscript. Each blot is labeled with the antibody that was used to probe the membrane, the size of the band detected, and each sample is labeled at the lane of the gel. Lanes with an X were not used. Each blot also indicates which parasite line was used for western blot analysis. (TIFF) [file pone.0334727.s006.tiff]
